# Ordered and Disordered Phases in Mo_1−x_W_x_S_2_ Monolayer

**DOI:** 10.1038/s41598-017-15286-9

**Published:** 2017-11-09

**Authors:** Wei Tan, Zhipeng Wei, Xiaomin Liu, Jialin Liu, Xuan Fang, Dan Fang, Xiaohua Wang, Dengkui Wang, Jilong Tang, Xiaofeng Fan

**Affiliations:** 1 State Key Laboratory of High Power Semiconductor Laser, Changchun University of Science and Technology, Changchun, 130022 China; 2 Key Laboratory of Automobile Materials (Jilin University), Ministry of Education, and College of Materials Science and Engineering, Jilin University, Changchun, 130012 China

## Abstract

With special quasirandom structure approach and cluster expansion method combined with first-principle calculations, we explore the structure and electronic properties of monolayer Mo_1−x_W_x_S_2_ alloy with disordered phase and ordered phase. The phase transition from ordered phase to disordered phase is found to happen at 41 K and 43 K for *x* = 1/3 and *x* = 2/3, respectively. The band edge of VBM is just related with the composition *x*, while the band edge of CBM is sensitive to the degree of order, besides the concentration of W. Near the CBM band edge, there are two bands with the Mo-character and W-character, respectively. It is found that in disordered phase the Mo-character band is mixed with the W-character band, while the opposite happens in ordered phase. This result leads to that the splitting of two bands near CBM in ordered phase is larger than in disordered phase and gives rise to the smaller band gap in ordered phase compared to the disordered phase. The electron effective mass in ordered phase is smaller than in disordered phase, while the heavy hole effective mass in ordered phase is larger than that in disordered phase.

## Introduction

The appearance of graphene in 2004^1^, has opened the door of studying atomically thin two-dimensional (2D) materials. The large 2D material family includes graphene, h-BN, transition metal dichalcogenides (TMDs), and so on^2,3^. These 2D materials exhibit the novel electronic and optical properties for the applications of versatile devices^4–6^. As an important member of 2D materials family, TMDs have attracted extensive attention owing to their sizeable band gaps^7–9^. TMDs share a common chemical formula MX_2_ where M is a transition metal of groups IVB,VB and VIB, and X is chalcogen of group VA. From the aspect of atomic structure, monolayer TMDs show a “sandwich” structure with a sheet of metal atoms sandwiched between two sheets of chalcogens. Interestingly, we can get different “taste” sandwich according to the atomic structures, such as 1 T phase (metallic or semiconducting property), and 2 H phase (semiconductor). These monolayer TMDs have been used for the applications within hydrogen evolution^10^, field effect transistors^11,12^, photodetectors^13^, etc. The present work puts eyes on 2 H phase semiconducting monolayer TMDs materials.

As the advance of the times with the development of technologies, devices with all kinds of properties are always needed to satisfy the requirements in daily life and industrial production^14^. Usually, a specific material only has its specific property. Alloying is one of ways used to modulate the property of materials, especially in semiconductors^15–18^. Recently, monolayer Mo_1−x_W_x_S_2_ has been synthesized experimentally^19–24^, with tunable band gap, band edge position and carriers’ effective mass^25^. In addition, Mo_1−x_W_x_S_2_ thin layer is applied in electrochemistry and possesses superior hydrogen evolution reactionperformance^26,27^.

In terms of alloy, when two end materials are mixed together, the most basic question is how these atoms are distributed, since atomic distribution plays a critical role in the properties of alloying materials. Scanning transmission electron microscopy (STEM) has demonstrated a disordered arrangement of Mo and W atoms in monolayer Mo_1−x_W_x_S_2_ throughout the chemical compositions from 0 to 1^19,24^. Quantitative analysis of the atomic distributions has been carried out in monolayer Mo_1−x_W_x_S_2_, and it has been found there is a disordered distribution for Mo and W^28^. From the theoretical part, the ordered monolayer Mo_1−x_W_x_S_2_ phase is predicted at x = 1/3 and x = 2/3^29^. Moreover, in the present experiment, a particular high temperature is used to fabricate monolayer Mo_1−x_W_x_S_2_.We all share the same opinion that different atomic distribution (ordered or disordered) of an alloying material has an effect on material properties. However, it is not clear that what the affected properties lies on and how the atomic distribution changes the properties of Mo_1−x_W_x_S_2_. Here we will focus on the comparison of ordered and disordered phase of monolayer Mo_1−x_W_x_S_2_ and provide a new choice for the need of devices with versatile properties based on 2D alloy materials.

In this paper, we compare the thermodynamic and electronic properties of the ordered and disordered phase in monolayer Mo_1−x_W_X_S_2_. We find that the energy of ordered phase is smaller than that of disordered phase at 0 K. With increasing temperature to 43 K or higher, disordered phase is easier to fabricate than ordered phase. The calculated energy bands reveal that the valence band maximum (VBM) is a two-fold degenerate band in both ordered and disordered phase with the same valence band edge, and with different behaviors near the conduction band minimum (CBM). The band gap of ordered phase is found to be smaller than disordered phase. The calculated electron effective mass in ordered phase is smaller than the disordered one, but the heavy hole effective mass of ordered phase is larger than the disordered one.

## Results and Discussion

### Thermodynamic properties

To explore the possible ordered phases in alloy Mo_1−x_W_x_S_2_ with single layer, cluster expansion (CE) method combined with supercell method is adopted. CE can analyze all the structures with different concentration *x* in configuration space. In a supercell with 2D lattice space, Mo and W atoms are settled with all possible ways in some small 2D cells to obtain the different configurations of alloy. Then the S atoms are added in these alloy configurations. In other words, by the way of W atoms replaced by Mo atoms in WS_2_ lattice with considering the replaced positions and quantity of replaced atoms, the different alloy’s structures with different *x* can be proposed. In addition, the size and shape of 2D lattice supercell are also considered. With these constructed atomic structures, first-principle calculations are preformed. With the calculated energies to fit the interaction parameters in the CE formula, the energies of other configurations can be predicted in a self-consistent way. In the calculations, the formation energy is defined by the formula,

1 $${E}_{{\rm{Form}}}(\{\sigma \},x)={E}_{{{\rm{Mo}}}_{1-x}{{\rm{W}}}_{x}{{\rm{S}}}_{2}}(\{\sigma \},x)\,-\,[x{E}_{{{\rm{WS}}}_{2}}+(1\,-\,x){E}_{{{\rm{MoS}}}_{2}}],$$

where ${E}_{M{o}_{1-x}{W}_{x}{S}_{2}}(\{\sigma \},x)$, ${E}_{Mo{S}_{2}}$ and ${E}_{W{S}_{2}}$ are the total energies of Mo_1−x_W_x_S_2_ with configuration {σ}, pure MoS_2_, and pure WS_2_, respectively.

**Figure 1:**
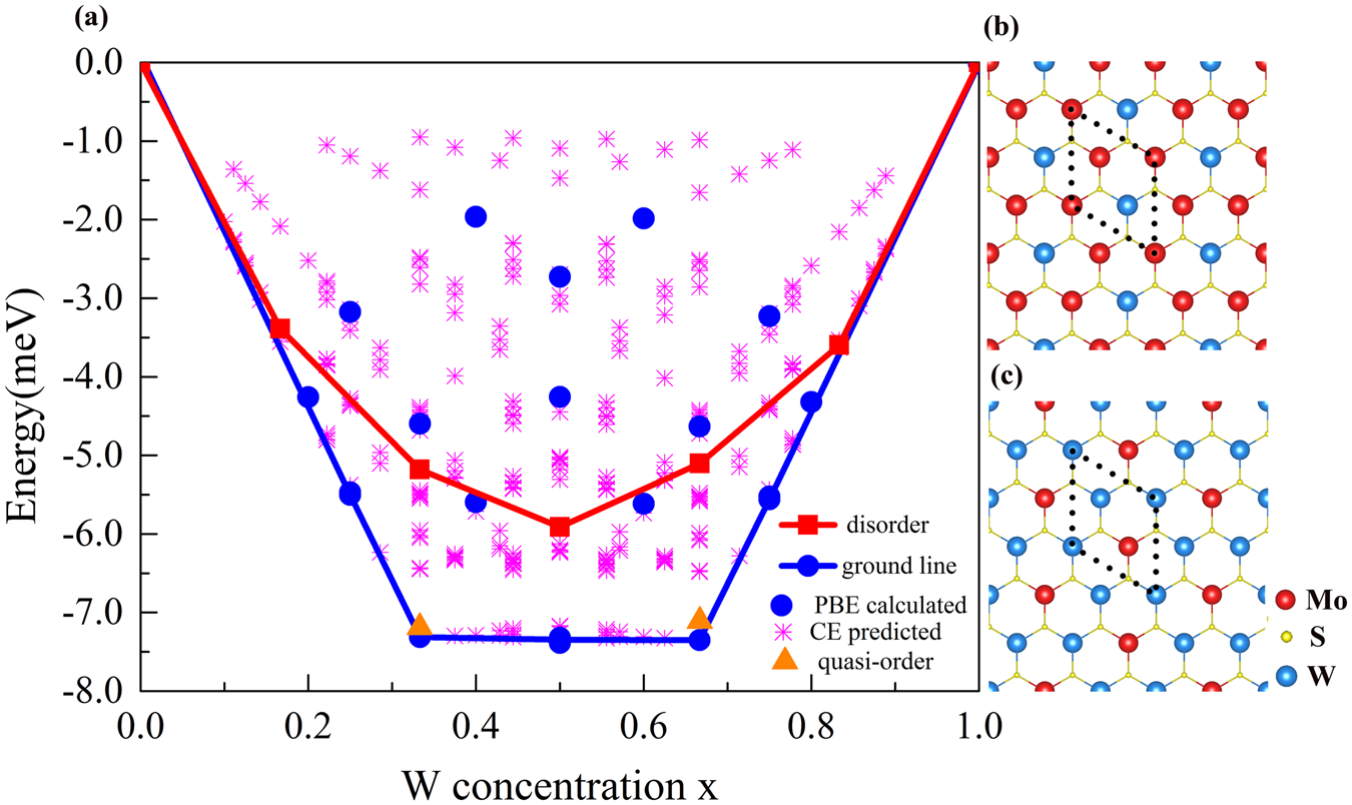
(**a**) Formation energies per formula cell of monolayer Mo_1−x_W_x_S_2_ and atomic structures of two ordered phases with (**b**) *x* = 1/3 and (**c**) *x* = 2/3. The blue dots represent the values calculated from DFT, and the pink stars represent the values from CE prediction. The blue solid line with dots is for the convex hull of ground states. The red solid with squares and orange triangles represent the values of disordered phase and quasi-ordered phase, respectively.

In Fig. 1a, we show the calculated formation energies of all the configurations considered and the convex hull of alloy ground states of monolayer Mo_1−x_W_x_S_2_ (in blue dot and line). The negative formation energies of all these alloy configurations suggest that monolayer Mo_1−x_W_x_S_2_ is stable at zero temperature. From the convex hull, two ordered alloy phases are found to be at *x* = 1/3 and 2/3, respectively. This is in good agreement with previous report^29^. The atomic structures of two phases are shown in Fig. 1b,c. It is found that the unit cell of two ordered phases is just with three metallic atoms and highly ordered for the arrangement of W and Mo.

From the formation energies in Fig. 1a, we propose the possibility of disordered phases. For the different *x*, there may be a lot of different structures about disordered phases. For same concentration of *x*, the band structures and formation energies of different structures about disordered phase should be similar. Here, the special quasirandom structure (SQS) method is used to construct the structure of disordered phase for each concentration x. Five SQS structures with the concentration of *x* = 1/6, 1/3, 1/2, 2/3 and 5/6 are proposed. The formation energies of five disordered phases are shown in Fig. 1a with red solid line with square. It is noticed that the formation energies of these disordered phases are negative. For two special compositions (*x* = 1/3 and 2/3), the formation energies of disordered phases are larger than that of ordered phase. This implies that ordered phase will be fabricated more easily than disordered phase at low temperature. However, during the growth of Mo_1−x_W_x_S_2_, the ordered phase is likely to be broken to form the so-called quasi-ordered phase due to the temperature effect. We construct a 3 × 3 × 1 supercell of the ordered phase, and then the position of one W atom is exchanged with one of its nearest neighbor Mo atoms (see Fig. S1 of SI). The formation energies of both quasi-ordered phases with *x* = 1/3 and 2/3 are shown with orange triangles in Fig. 1. It can be seen that the formation energies of quasi-ordered phases area are a little larger than that of ordered phases, but smaller than disordered phases. It is be possible the addition of external factors, such as temperature, will break the order arrangement of atoms and make the conversion from the ordered phase to the disordered.

In present practical growth of monolayer Mo_1−x_W_x_S_2_, the growth temperature is controlled between 700–1000 °C^19–24^. The difference of temperature depends mainly on growth approach. Here, the change of free energy of alloy with temperature is explored by considering the configuration effect. The contributions of phonons and electrons for the difference of the formation energy between disordered phase and ordered phase are considered to be small and ignored. The free energy of alloy is defined by the formula,

2 $$F(x)={E}_{{\rm{Form}}}(x)\,-\,TS(x),$$

where *E*_Form_(*x*) is the formation energy of ordered/disordered phase with the composition of *x*. T and *S*(*x*) represent the temperature and entropy, respectively. The alloying entropy of disordered phase from configurations can be expressed by the formula^30^,

3 $$S(x)=-{k}_{B}[x\,{\rm{ln}}\,x+(1\,-\,x){\rm{ln}}(1\,-\,x)].$$

**Figure 2:**
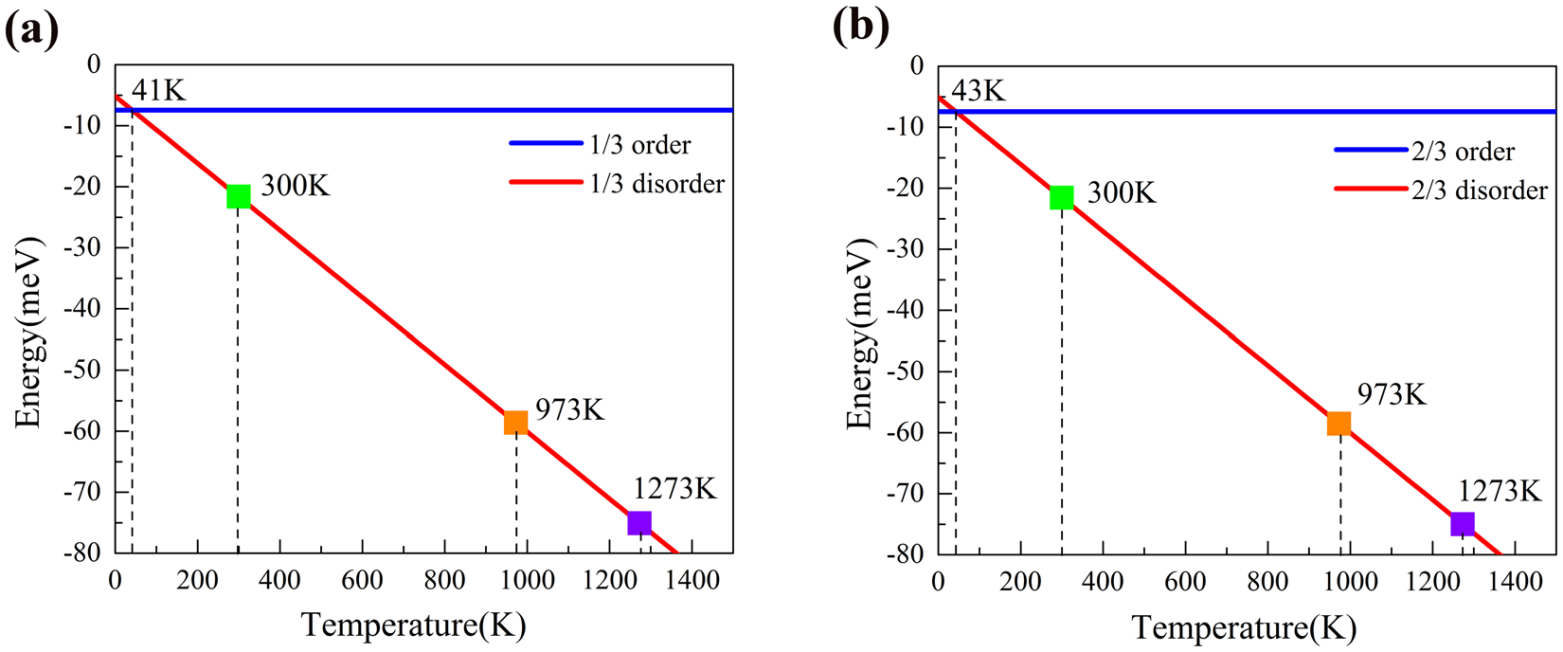
Free energies of ordered phases and disordered phases (*x* = 1/3 and 2/3) as a function of temperature, at the composition of (**a**) *x* = 1/3, and (**b**) *x* = 1/3. The blue solid and red solid denote the phases of order and disorder, respectively. Four particular dots are shown for the free energies at temperature, 41 K, 300 K, 973 K and 1273 K.

Obviously, the alloy entropy of ordered phase is zero and its free energy remains unchanged with the increasing of temperature. Figure 2a shows the evolution of free energy as a function of the temperature at concentration *x* = 1/3. For the disordered phase, the free energy decreases as temperature increases, intersecting with that of ordered phase at 41 K. It means the disordered phase becomes to be more stable than ordered phase due to the contribution of entropy, by following the increase of temperature. The phase transition temperature from the ordered to the disordered is 41 K. the disordered phase will be fabricated more easily than ordered phase. When the temperature increases to 300 K, 973 K, and 1273 K, the free energies of disordered phase are −21.58 meV, −58.63 meV, and −72.28 meV, respectively. This is the reason why monolayer Mo_1−x_W_x_S_2_ was observed to be with the disordered distribution of W and Mo in recent experiments with a particular high growth temperature. Figure 2b exhibits the curve of free energy with concentration *x* = 2/3. The trend of disordered phase is the same as *x* = 1/3, and the phase transition temperature is 43 K. When the temperature increases to 300 K, 973 K, and 1273 K, the free energy of disordered phase will decrease to −21.58 meV, −58.55 meV, and −75.03 meV, respectively.

### Energy band structures of Mo_1−x_W_x_S_2_

**Figure 3:**
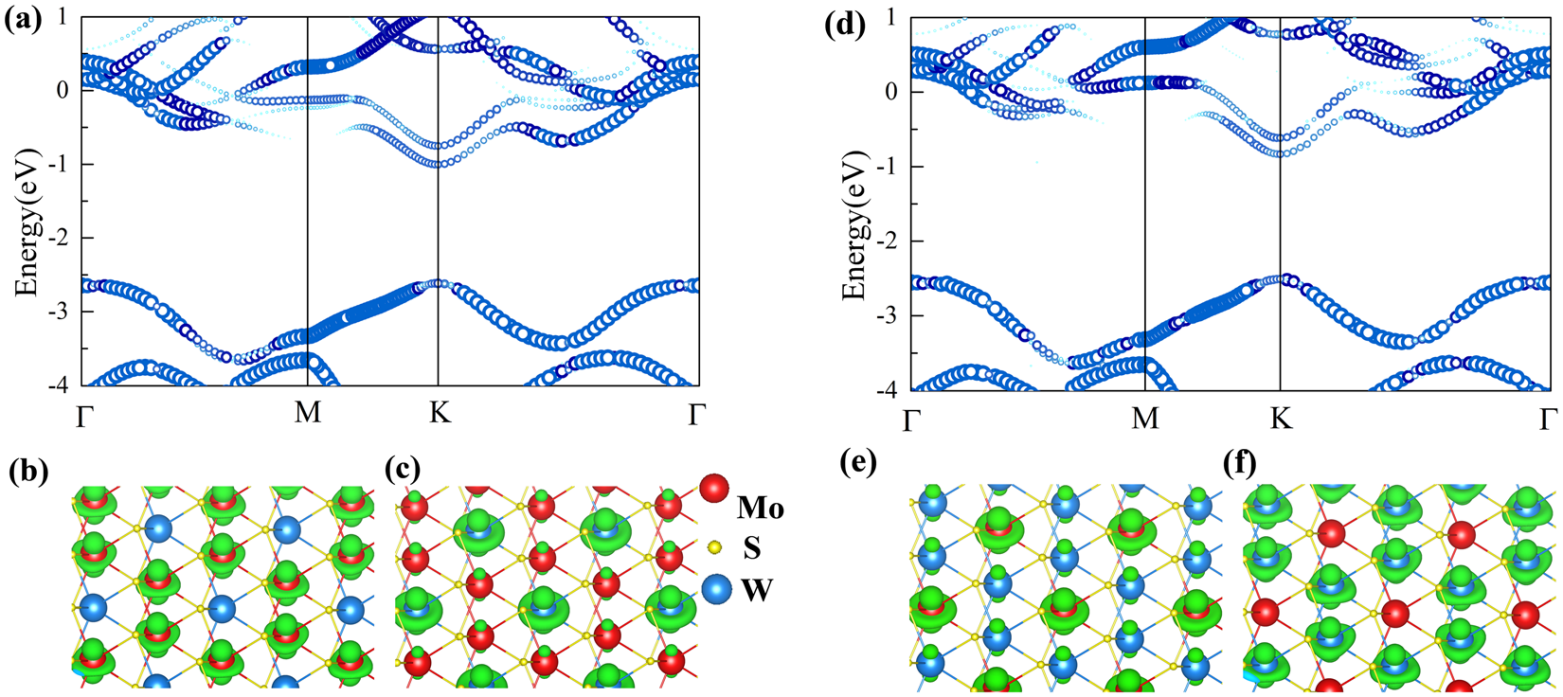
Unfolded energy band of ordered phases with (**a**) *x* = 1/3 and (**d**) *x* = 2/3, and the corresponding real space charge distributions of two bands near CBM at K point, for (**b**,**c**) *x* = 1/3 and (**e**,**f**) *x* = 2/3. The lower-energy band in two bands is with the Mo-character (**b**,**e**), and the higher-energy is with the W-character (**c**,**f**). The red, blue and yellow atoms represent Mo, W, and S, respectively.

It is a common practice to use supercell method with first-principle calculations in the study of alloy. As the supercell becomes larger, the corresponding Brillouin zone shrinks and the calculated band structure becomes dense due to the band folding. It is usually hard to deduce useful information from such heavily folded energy bands. With QU method, the bands can be unfolded into the Brillouin zone of primary cell. From the folded energy band (see Figs S2 and S3 of SI), VBM and CBM of ordered phase (monolayer Mo_1−x_W_x_S_2_ with *x* = 1/3 and 2/3) are located at Γ point of Brillouin zone. The VBM is a two-fold degenerate band at Γ. Figure 3a,d show the unfolded bands in the first Brillouin zone of ordered phases (*x* = 1/3 and 2/3), where the shade of dots represents the weight of each eigenvalues. The unfolded energy bands of alloy have broken points and darkness in a variety, which originates from the breaking of transitional symmetry. The VBM and CBM of monolayer Mo_1−x_W_x_S_2_ are located at K point of Brillouin zone, similar to MoS_2_ and WS_2_. The VBM at K is a two-fold degenerate band, and for the CBM at K there is a clear neighboring band. It is deduced that the CBM and its neighboring band at Γ in the folded band structure (Fig. S2 of SI) are unfolded into the K point in the unfolded band structure from primitive cell.

The real space distributions of charge densities of CBM and its neighboring band at K point are calculated to analyze the origin of these bands. In Fig. 3b,c, the charge densities at CBM and its neighboring band of ordered phase with *x* = 1/3 are shown. The charges of CBM at K only distribute at Mo atoms and the state at CBM has dz^2^ characteristic. Its neighboring band at K is contributed mainly by the orbitals from W atoms with the same dz^2^ characteristic. Figure 3e,f show the charge densities at CBM and its neighboring bands of the ordered phase with *x* = 2/3. The charge density of CBM also distributes mainly at Mo atoms with dz^2^ characteristic. Its neighboring band is only contributed by W atom. It is well known that the CBM of both WS_2_ and MoS_2_ are dominated by dz^2^ orbitals of cations. The energy of 5dz^2^ orbital of WS_2_ is higher than 4dz^2^ orbital of MoS_2_. So the CBM and its neighboring band of ordered phase (*x* = 1/3 and 2/3) are dominated by Mo and W atoms, respectively. We name CBM as the Mo-character band, and its neighboring band as W-character band. Namely, when pure MoS_2_ and pure WS_2_ are mixed together as Mo_1−x_W_x_S_2_, the Mo-character band and W-character band are appeared at CBM and its neighboring band, respectively. The two-fold degenerate band at K of VBM observed which mainly from the orbitals dx^2^−y^2^ and dxy is because of the same contribution to VBM of WS_2_ and MoS_2_.

**Figure 4:**
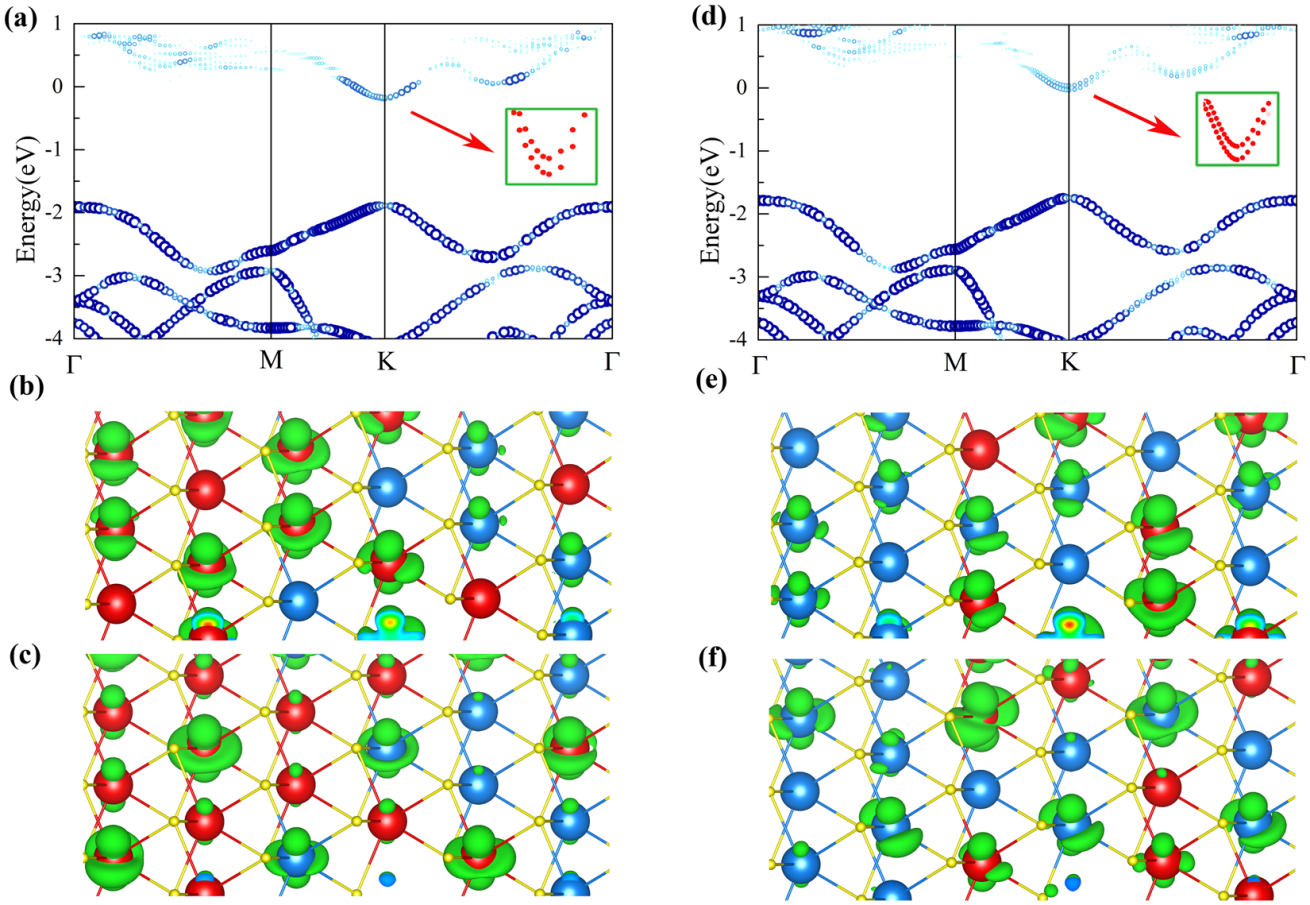
Unfolded energy band of disordered phases with (**a**) *x* = 1/3 and (**d**) x = 2/3, and the corresponding real space charge distributions of two bands near CBM at K point, for (**b**,**c**) *x* = 1/3 and (**e**,**f**) *x* = 2/3. The lower-energy band in two bands is with the Mo-character (**b**,**e**), and the higher-energy is with the W-character (**c**,**f**) to some extent.

The energy bands of disordered phases (*x* = 1/3 and 2/3) are shown Fig. 4a,d. Similar to the energy band of ordered phase, the two-fold degenerate VBM at Γ point in the folded band (Fig. S3 of SI) is unfolded into K point in the unfolded band structure. We also observe the two bands near CBM (see the insets in Fig. 4a,d) with the Mo-character and W-character band, but compared with the two bands near CBM at K in ordered phase, the energy difference between the two bands is small. The energy difference in disordered phases are about 20 meV and 80 meV for *x* = 1/3 and *x* = 2/3, respectively. In ordered phases with *x* = 1/3 and *x* = 2/3, the energy difference between two bands near CBM at K are 220 meV and 250 meV, respectively. The charge densities of two bands near CBM at K are shown in Fig. 4b,c,e and f for both *x* = 1/3 and *x* = 2/3. In two bands near CBM at K, the lower-energy band (CBM) is mainly with the Mo-dz^2^ characteristic and the higher-energy band is mainly with the W-dz^2^ characteristic. But due to the disordered arrange of W and Mo in disordered phase, some dz^2^ orbtials from W atoms are mixed into the lower-energy band and some dz^2^ orbtials from Mo atoms are mixed into the higher-energy band near CBM at K. This may be the reason why the band splitting near CBM at K in disordered phase is smaller than in ordered phase.

### Band gap of Mo_1−x_W_x_S_2_

**Figure 5:**
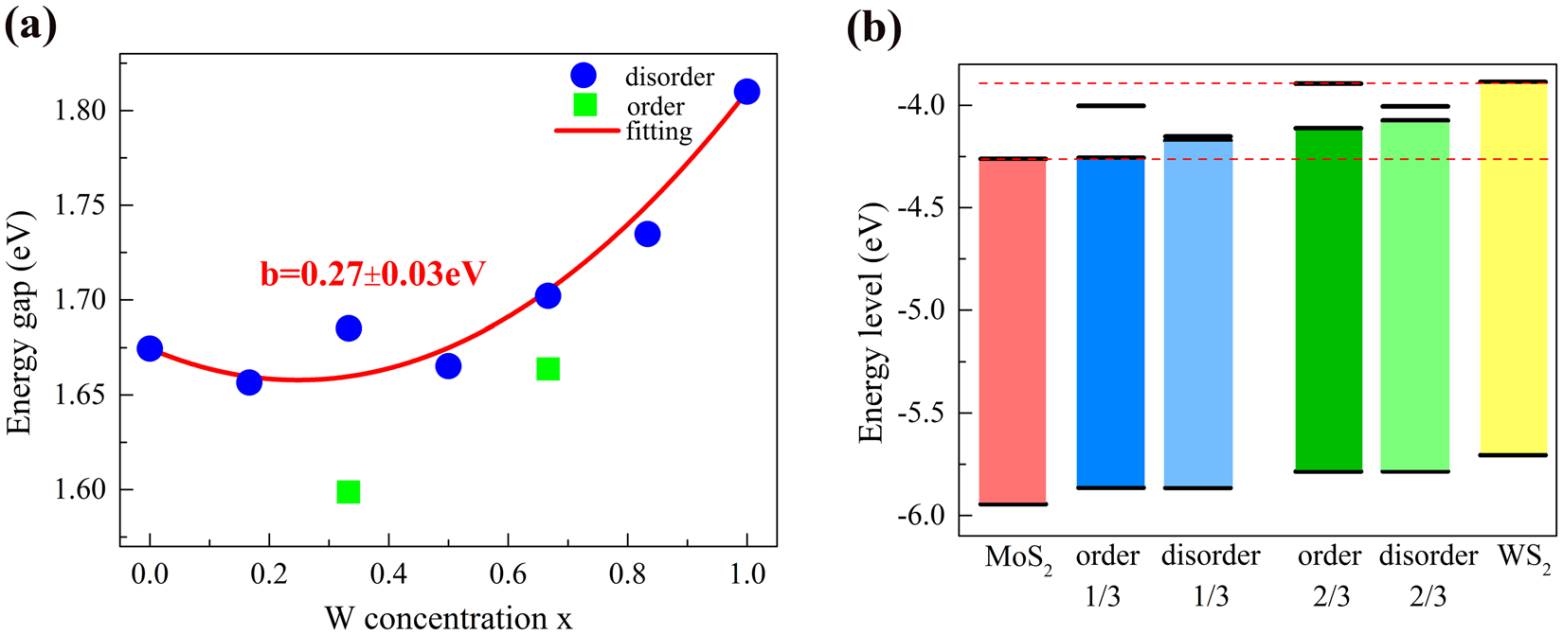
(**a**) Electronic band gap of disordered phases at all the concentrations and ordered phase with *x* = 1/3 and 2/3. The circle and square represent the disordered and ordered phase, respectively. The parabola represented by red solid line is from the results by fitting the data of disordered phases. (**b**) Band edge positions of MoS_2_, WS_2_, ordered phases and disordered phases relative to the vacuum level. In alloys with the ordered and disordered phases, the two bands near CBM with the Mo-character and W-character are exhibited.

As we all know, band gap of an optoelectronic material is vital for its potential applications. We extract the band gaps from the band structures of both ordered phase and disordered phase. It is noticed that the spin-orbit coupling (SOC) effect leads to a significant splitting at VBM of K point in MoS_2_ and WS_2_^31–33^. Here SOC is not considered in the calculations because the band gap of WS_2_ with SOC is smaller than that of MoS_2_ and inconsistent with the experimental results^19^. In addition, SOC doesn’t change the trend of effective mass and the band character near CBM and VBM in MoS_2_ and WS_2_^25^. In Fig. 5a, we present the band gap as a function of concentration *x*. It can be found that the band gap of monolayer Mo_1−x_W_x_S_2_ with disordered phase exhibits a nonlinear to the composition *x* with the bowing effect, as the observation in most 3D semiconductor alloys. The band gap bowing can be described by the formula,

4 $${E}_{g}(M{o}_{1-x}{W}_{x}{S}_{2})=(1\,-\,x){E}_{g}(Mo{S}_{2})+x{E}_{g}(W{S}_{2})\,-\,bx(1\,-\,x),$$

where *b* is the so-called bowing parameter. By fitting the curve, the bowing parameter *b* obtained is 0.27 ± 0.03 eV, in good agreement with experimental value of 0.25 ± 0.04 eV^9^. In Fig. 5a, the band gaps of ordered phases (*x* = 1/3 and 2/3) are also presented with the green squares. Obviously, the band gap of ordered phase is smaller than disordered phase for each composition of W.

To understand the variation of band gap, the absolute energy levels including VBM and CBM with the Mo-character and W-character in ordered phase and disordered phase (*x* = 1/3 and 2/3), and that in MoS_2_ and WS_2_, are determined by the method of vacuum level calibration^34^. As shown in Fig. 5b and Fig. S5 of SI, with the increase of the composition *x*, the energy levels of VBM and CBM increase nonlinearly from MoS_2_ to WS_2_. This results in the bowing effect mostly due to charge exchange with different electronegativity of W and Mo and local structure relaxation. Interestingly, for the same concentration whether *x* = 1/3 or 2/3, the VBM band edges of the ordered, quasi-ordered (Fig. S5 of SI), and disordered phase are same. This implies that the VBM band edge is just related with the composition *x* and insensitive to the arrange order of Mo and W in the lattice. This can be explained by the same orbital contribution of Mo and W, and the delocalization effect of orbitals near VBM.

In the CBM, there are some different behaviors from VBM for the structures with same composition *x*. For the ordered phase with *x* = 1/3, the band edge of Mo-character band is consistent with the CBM band edge of MoS_2_.The band edge of W-character band moves down from the CBM band edge of WS_2_ in Fig. 5b. This may be due to the lower VBM band edge of ordered phase with *x* = 1/3 than WS_2_. For the disordered phase with *x* = 1/3, the low-energy Mo-character band and high-energy W-character band are away from the CBM of MoS_2_ and CBM of WS_2_, respectively. This leads to the smaller splitting of both bands than in the ordered phase. This may be explained by the unique charge distribution in ordered phase with high symmetry which results in the separation of orbitals from Mo-dz^2^ and W-dz^2^. In disordered phase with the breaking of local symmetry, the orbitals from Mo-dz^2^ are hybridized with that from W-dz^2^, as the discussion about the charge distributions above. This is confirmed by the band splitting near CBM of quasi-ordered phase (Fig. S2 of SI). The position exchange between one of Mo atoms and W from ordered phase results in the weaker hybridization which leads to the decrease of band splitting compared with the ordered phase. Similar phenomena happen in the phase with *x* = 2/3. In the ordered phase with *x* = 2/3, the lower-energy Mo-character band deviates from CBM of MoS_2_ due to the up-shift of VBM and the energy level of higher-energy W-character band is equal to CBM of WS_2_. We also calculate the band edge of disordered phase with W concentration (*x* = 1/6, 1/2 and 5/6 in Fig. S4 of SI), which is consistent with the previous work^25^. Therefore, the larger splitting of both bands near CBM in ordered phases results in its smaller band gap than that of disordered phase. In addition, we can propose that the bowing parameter will increase by following the increase of the degree of order, as the observation in ZnO_*x*_S_1−x_ alloy^35^.

### Carriers’ effective mass

**Figure 6:**
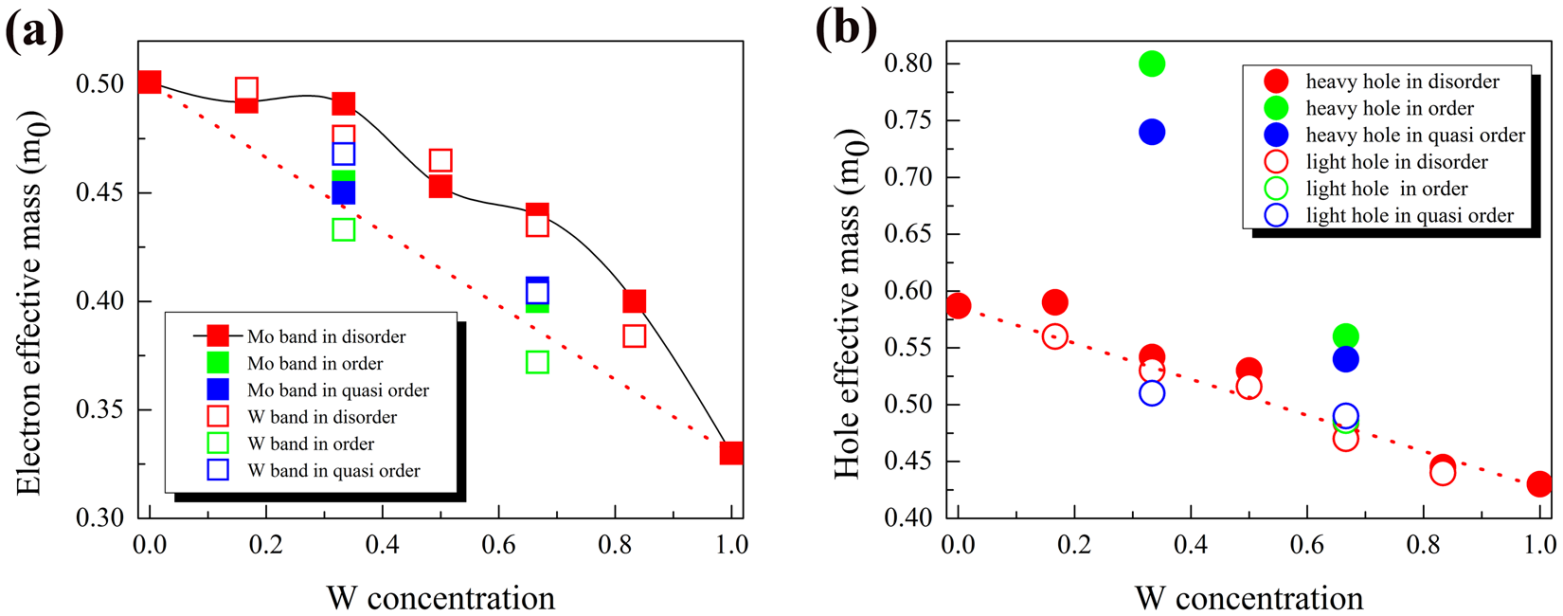
Effective masses of (**a**) electrons and (**b**) holes for monolayer Mo_1−x_W_x_S_2_. In (**a**) the red, green, blue filled and open squares represent the effective masses of electrons with the Mo-character and W-character bands in the disordered, ordered, and quasi-ordered phase respectively. In (**b**), the red, green, blue filled and open circles represent the effective masses of heavy holes and light holes in disordered, ordered, and quasi-ordered phases, respectively.

We proceed with the calculation of the effective carrier mass at VBM and CBM in ordered and disordered phase to explore the transport properties. The effective mass of carriers is given by the formula, $\frac{1}{{m}^{* }}=\frac{1}{{\hslash }^{2}}\frac{dE(k)}{d{k}^{2}}$. Figure 6a,b show the calculated effective mass of electrons and holes in the ordered, quasi-ordered and disordered phase. As the discussion above, there are the lower-energy Mo-character band and higher-energy W-character band near the CBM. As shown in Fig. 6a, for the Mo-character band in disordered phase, the effective mass of electron varies with W concentration nonlinearly, which is in agreement with the trend reported in the literatures^25^. The electron effective mass of the W-character band has the similar trend, following the change of W concentration. In addition, it is found that in the quasi-ordered and ordered phase (*x* = 1/3 and 2/3), the electron effective mass is smaller than in the disordered phase, whatever the Mo-character band or W-character band. The smaller effective mass of electrons and larger difference of the masses from two bands (Mo-character and W-character) are probably because of the larger energy splitting between two bands in ordered phase. In disordered phase shown in Fig. 6b, the effective mass of light hole is a little smaller than the heavy hole and both decrease almost linearly with the increase of W concentration. For the ordered and quasi-ordered phase (x = 1/3 and 2/3), the effective mass of light hole is similar to the disordered phase. Interestingly, it is found that the effective masses of heavy holes are very larger.

## Conclusions and Outlook

We study the thermodynamic and electronic properties of ordered and disordered phases in monolayer Mo_1−x_W_x_S_2_ alloy by first-principle calculations combined with SQS method, CE method and quantum unfolding about electronic structures. It is found that there is a phase transition from the ordered phase to disordered phase at the composition *x* = 1/3 and 2/3 following the increase of growth temperature. The phase transition temperatures are 41 K and 43 K, respectively. This is attributed mainly to the contribution of configuration entropy due to the disordered arrange of W and Mo.

From the electronic structures of disordered phases, there is an obvious bowing effect for the band gap with the bowing parameter b = 0.27 ± 0.03 eV, consistent with experimental value. The energy band shows that there is a two-fold degenerate band in VBM in the alloy. The VBM band edge is just correlated with the composition *x* and insensitive to the degree of disorder from the disordered arrange of W and Mo. However, the CBM band edge is very sensitive to the degree of disorder. We found there are two energy bands near CBM with the Mo-character and W-character, respectively. In the ordered phase, the lower-energy Mo-character band is decoupled with the higher-energy W-character band due to the high local symmetry. In the disordered phase, the two bands are mixed with each other and results in the smaller splitting of two bands, compared with the ordered phase. This also results in that the band gap of ordered phase is smaller than that of disordered phase. The calculated electron effective mass of disordered phase is larger than ordered phase, while the effective mass of heavy hole in ordered phase is found to be very larger. These findings in the Mo_1−x_W_x_S_2_ alloy are expected to extend to the other 2D semiconductor alloys and call for further experiments for verification.

## Methods

To simulate the ideal disordered alloy Mo_1−x_W_x_S_2_, we construct five special quasirandom structures, following the change of W concentrations (*x* = 1/6, 1/3, 1/2, 2/3 and 5/6) in the $3\times 3\surd 3\times 1$ rectangle supercell using the special quasirandom structure (SQS) method^36,37^. The correlation functions about Mo and W in the constructed SQS are chosen to be close to those of an ideal disordered alloy. Therefore, it is expected that the physical properties of disordered alloy can be well described. The structures of ordered phases with W concentrations (*x* = 1/3 and 2/3) are obtained by exploring the configurations space with cluster expansion (CE) method^38,39^ implanted in ATAT code^40^ combined with fist-principle calculations. The atomic structures obtained are shown in Fig. S1 of SI.

All the first-principle calculations throughout this work are performed by the projector augmented wave potentials method based on density functional theory (DFT) implemented in Vienna Ab Initio Simulation Package (VASP) code^41,42^. We apply the generalized gradient approximation (GGA) with the parametrization of Perdew-Burke- Ernzerhof (PBE)^43^ to describe the exchange correlation interactions of electrons. The *k*-space integral and plane-wave basis are chosen to ensure that the total energy is converged. The convergence criterion for the self-consistent field energy was set to be 10^−6^eV. For the plane wave expansion, the kinetic energy cutoff of 450 eV is sufficient. The Monkhorst-Pack method is used to sample the *k*-points in the Brillouin zone^44^. The *k*-meshes of Γ-centered 9 × 5 × 1 and 7 × 7 × 1are used for disordered phase and ordered phase, respectively. In the supercell method, we apply the vacuum slab of 18 Å to remove the spurious interactions between neighboring layers along *z* direction. The lattice vectors and atom coordinates are fully relaxed until the forces are below 0.01 eV/Å. We use quantum unfolding (QU) method to unfold the band structure of alloy from supercell method into the fist Brillouin zone of primitive cell with QU band unfolding code^45,46^.

## Electronic supplementary material

Supplementary information
