# Supplementary material for: Ordered and Disordered Phases in Mo1−xWxS2 Monolayer
Source: Sci Rep. 2017 Nov 9;7:15124. doi: 10.1038/s41598-017-15286-9 (PMC5680180; doi:10.1038/s41598-017-15286-9)
Supplement: Supplementary file 1 — Supplementary information [file 41598_2017_15286_MOESM1_ESM.pdf]

Supplementary Information

Ordered and Disordered Phases in $Mo_{1-x}W_xS_2$ Monolayer

Wei Tan[a], Zhipeng Wei[a,*], Xiaomin Liu[a], JialinLiu[b], Xuan Fang[a], Dan Fang[a], Xiaohua Wang[a], Dengkui Wang[a], Jilong Tang[a], Xiaofeng Fan[b, †]

[a] *State Key Laboratory of High Power Semiconductor Laser, Changchun University of Science and Technology, Changchun 130022, China*
[b] *Key Laboratory of Automobile Materials (Jilin University), Ministry of Education, and College of Materials Science and Engineering, Jilin University, Changchun, 130012, China*

**,†Correspondence and requests for materials should be addressed, xffan@jlu.edu.cn (X.Fan); zpweicust@126.com (Z. Wei)*

Figure S1

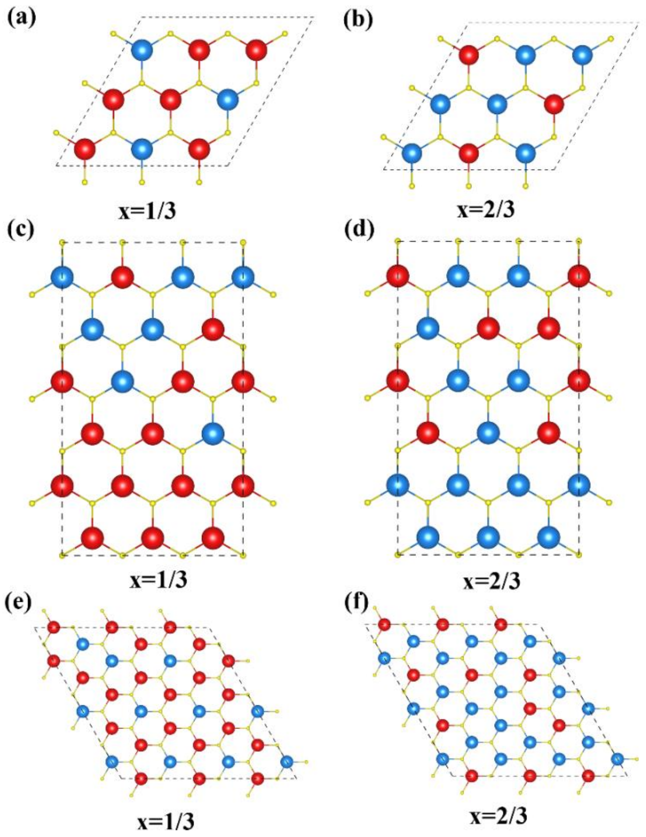

Figure S1. Structures of (a, b) the ordered phase, (c, d) disordered phase, and (e, f) quasi-ordered phase.

Figure S2

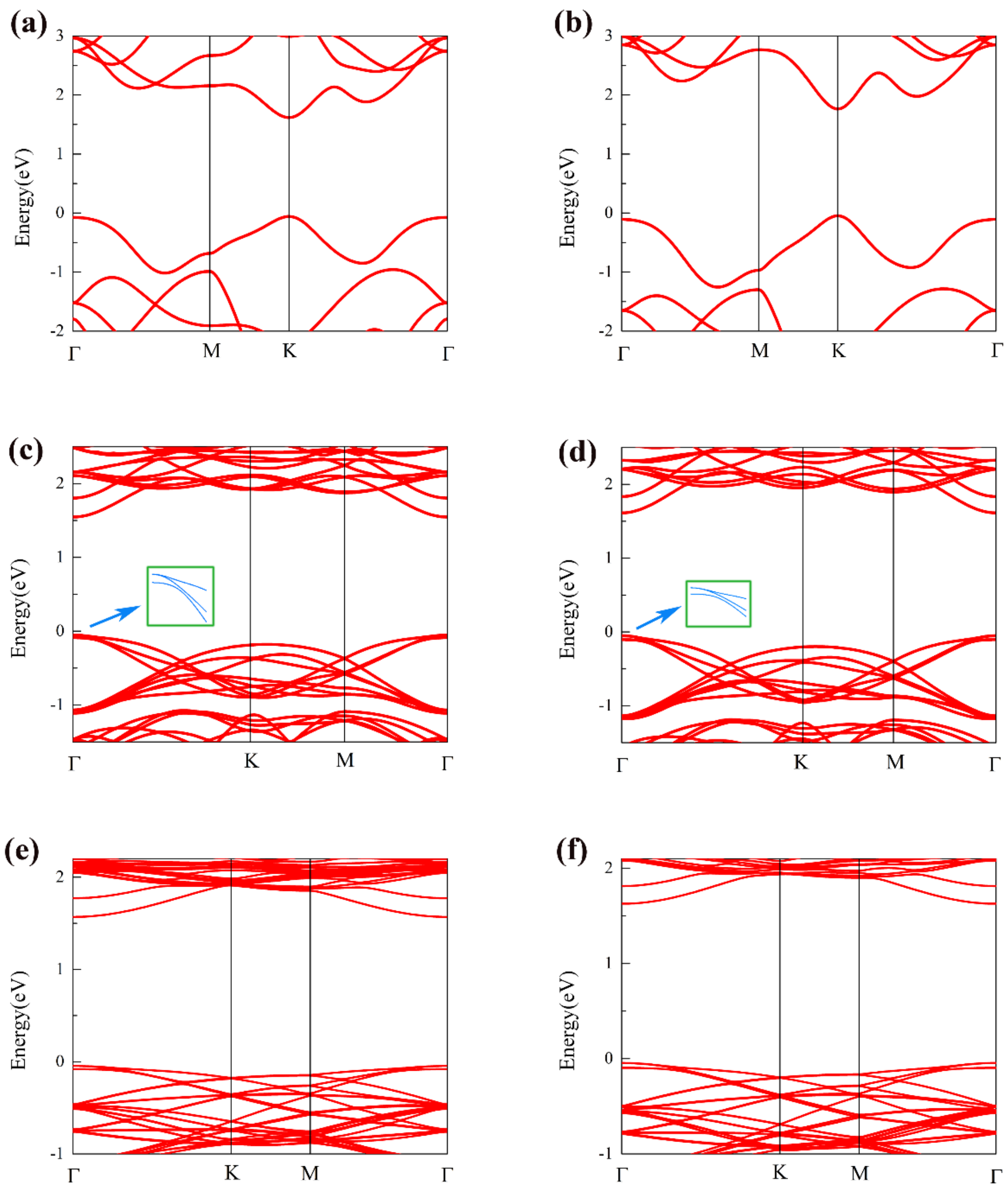

Figure S2. The folding energy band structures with supercell method for (a) MoS_2, (b) WS_2, (c) the ordered phase with x=1/3, (d) the ordered phase with x=2/3, (e) the quasi-ordered phase with x=1/3, and (f) the quasi-ordered phase with x=2/3. Note that the insets in (e, d) show the enlarged energy bands near the VBM atΓ.

Figure S3

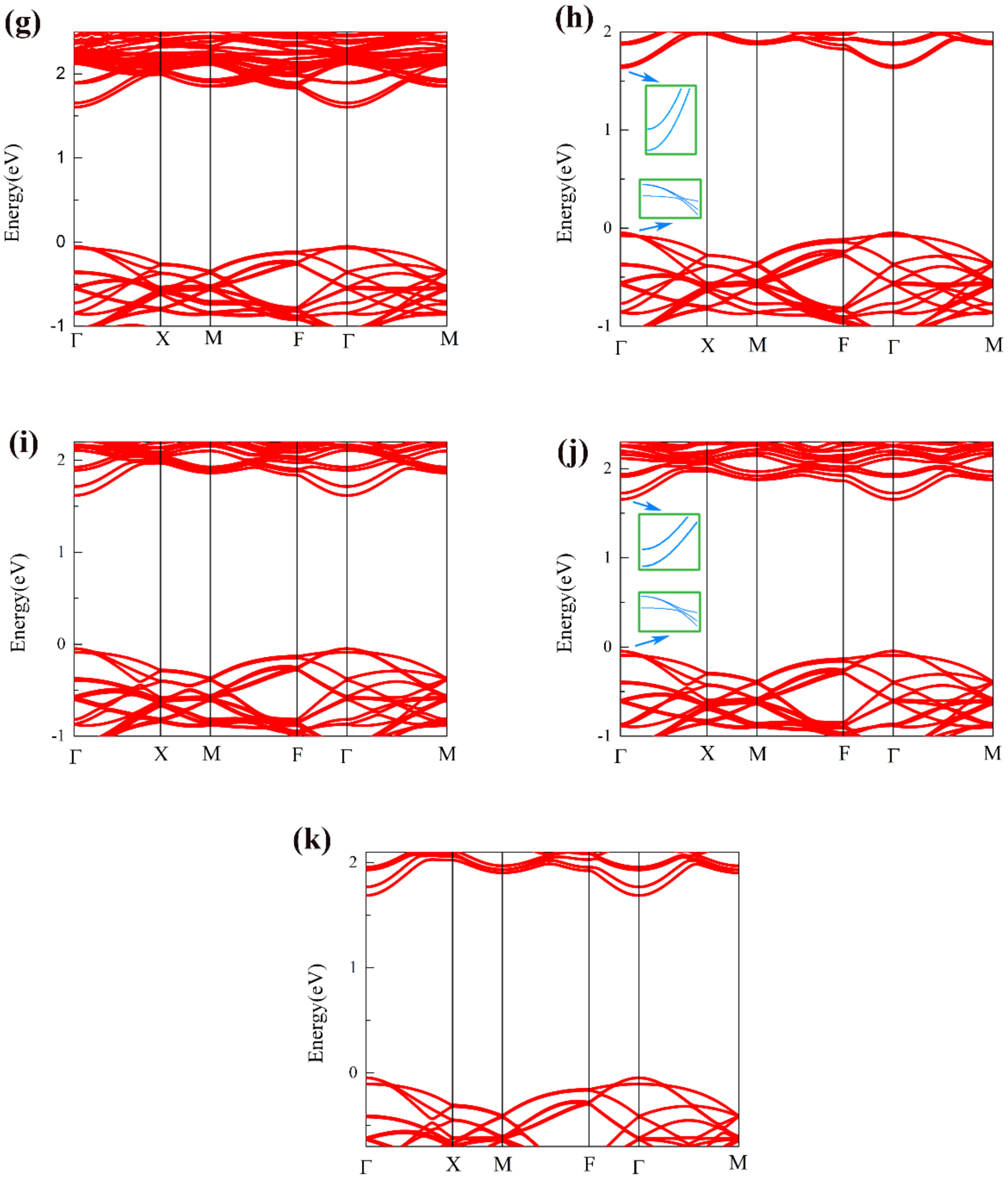

Figure S3. The folding energy band structures with supercell method for (g) the disordered phase with x=1/6, (h) the disorder phase with x= 1/3, (i) the disorder phase with x= 1/2, (j) the disorder phase with x=2/3, and (k) the disorder phase with x=5/6. Note that the insets in (h, j) show the enlarged energy band near the VBM and CBM atΓ.

Figure S4

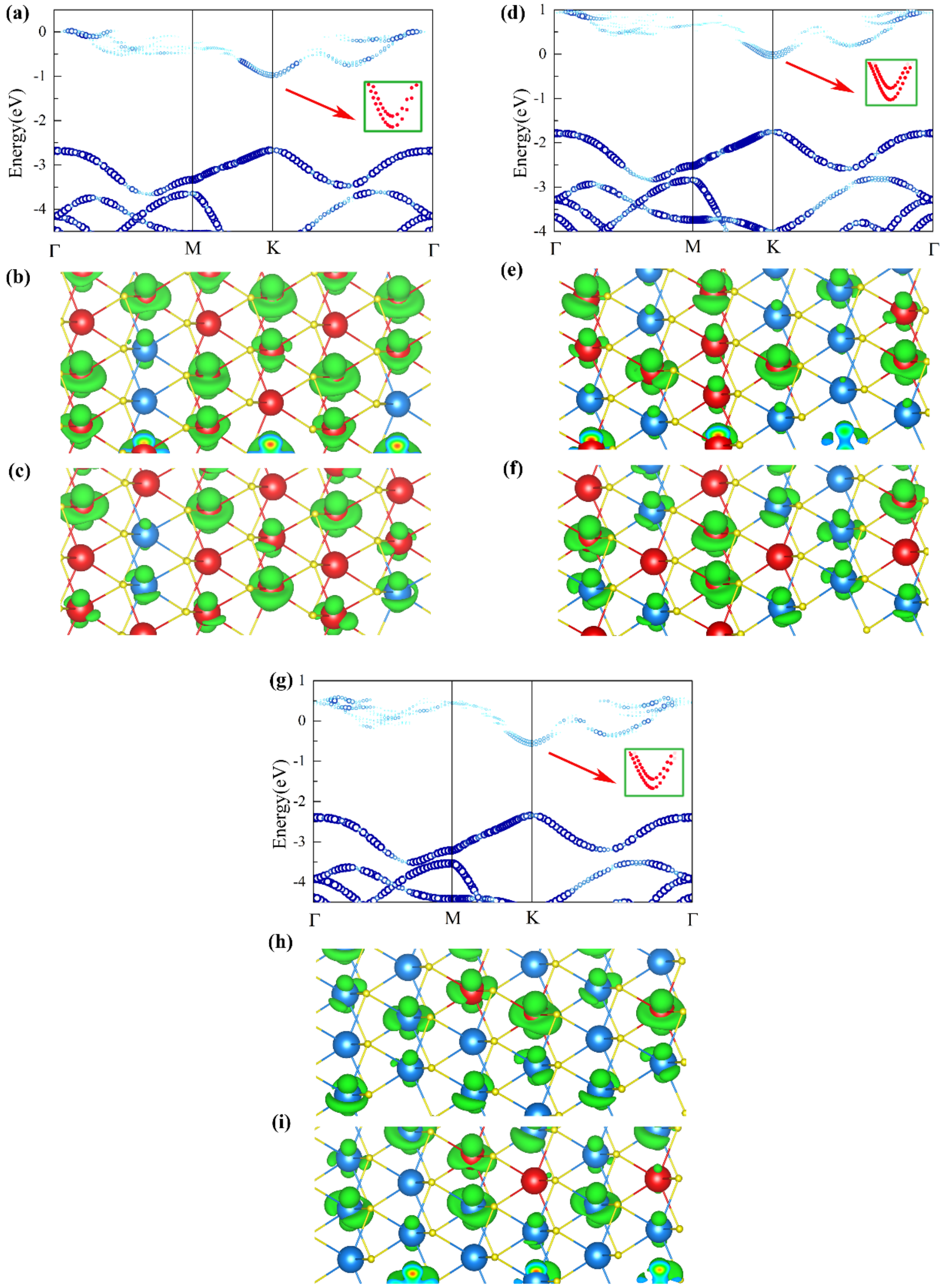

Figure S4. Unfolded energy band and corresponding charge density of Mo-character band and W-character band at K point in the disorder phase with (a, b and c) x=1/6, (d, e, and f) x=1/2, and (g, h, and i) x=5/6. Note that the inset shows the enlarged energy bands near the CBM at K.

Figure. S5

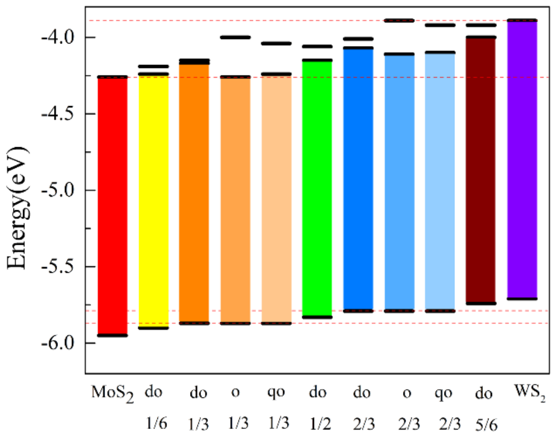

Figure S5. Band edge positions relative to vaccum level. Note that the "do", "o", and "qo" designate the disordered, ordered and quasi-ordered phase, respectively. From left to right, these bands are from MoS_2, disordered phase with x=1/6, disordered phase with x=1/3, ordered phase with x=1/3, quasi-ordered phase with x=1/3, disordered phase with x=1/2, disordered phase with x=2/3, ordered phase with x=2/3, quasi-ordered phase with x=2/3, disordered phase with x=5/6, and WS_2, respectively.
